# The Demand and Supply of Eunuchs in China

**Published:** 1897-02

**Authors:** 


### THE DEMAND AND SUPPLY OF EUNUCHS IN CHINA.

A writer in the London *Lancet* gives us some information on the above subject. In China the Emperor and certain members of the royal family are alone entitled to keep eunuchs. His majesty maintains at least 3,000, but no prince of the blood or imperial princess has a right to more than thirty. Theoretically, the palace eunuchs are furnished by governors of provinces, each of whom has to supply eight every five years, receiving in return 250 taels per eunuch. It was found, however, that the number thus obtained was totally insufficient, so a recruiting office was established at Pekin for the direct enrollment of candidates. In the production of Chinese eunuchs four chief factors prevail, viz., greed, predilection, poverty and laziness. Many parents sell their male children to the mutilators, or themselves castrate them in the hope of eventually sharing their earnings.

Young men of from twenty-five to thirty years of age, some of them having wives and families, often accept emasculation, being allured by the prospects of emolument. Poor wretches destitute of means and threatened with starvation agree to become eunuchs in order to gain a living. Finally, a certain number of lazy, good-for-nothing vagabonds sacrifice their manhood to secure a life of indolence. The operation is performed in a building situated close to one of the palace gates, but the operator, although his office is recognized and is a hereditary one, having been for many years in the same family, receives no regular wages, being entitled to a fee of six taels for each individual operated on. In the case of destitute candidates he exacts a lien on their prospective earnings. Dr. Matignon's description of the operation is as follows: The subject, with his abdomen and thighs tightly bandaged, is placed supine on a low bed, one assistant tightly grasping him around the waist, while two more keep his legs widely separated. The operator, as a rule, uses a curved implement resembling a pruning knife, but occasionally he substitutes for it a long pair of scissors. With his left hand he seizes the parts, squeezing and twisting them to diminish the supply of blood; but before cutting he inquires for the last time whether or not the patient is a consenting party. Adults, of course, answer for themselves, no anesthetic being used, but in the case of children the parents' word is accepted. The reply being in the affirmative, a single rapid sweep of the hand serves to remove both penis and scrotum, the blade of the instrument passing as close as possible to the pubis. A small piece of wood or of pewter, shaped like a nail, is then inserted into the urethra; the wound is washed two or three times with pepper and water and, several sheets of paper having been applied to the raw surface, the parts are carefully and tightly bandaged. The subsequent treatment is remarkable. Immediately after the bandaging the unfortunate patient is seized by the assistants and made to walk up and down the room at a rapid rate, not being permitted to lie down for three hours. For three days he is not allowed to drink anything, and not only does he suffer the pangs of thirst, but also has to endure the agonies of retention, owing to the plug in the urethra. On the fourth day the bandages are removed and the wretched creature is suffered to pass urine if he can. If the urine flows he is looked upon as cured, but should the overstrained bladder refuse to act he is left to die, the virtues of catheterization being apparently unknown to the Chinese. The amputation leaves a large triangular wound, with the apex downward, which takes on an average about a hundred days to granulate. Notwithstanding the primitive mode of procedure, the operation is usually successful, and fatal cases do not amount to more than three or four per cent. The most frequent complication is the incontinence of urine, but if this unpleasant symptom continues beyond a reasonable period the patient is condemned to flagellation, a mode of treatment which is said to yield the most excellent results.—*Journal A. M. A.*

